# Prospects for meteotsunami detection in earth’s atmosphere using GNSS observations

**DOI:** 10.1007/s10291-023-01492-8

**Published:** 2023-07-12

**Authors:** Panagiotis Vergados, Siddharth Krishnamoorthy, Léo Martire, Sebastijan Mrak, Attila Komjáthy, Yu T. Jade Morton, Ivica Vilibić

**Affiliations:** 1 Jet Propulsion Laboratory, California Institute of Technology, Pasadena, CA USA; 2 University of Colorado Boulder, Boulder, CO USA; 3 Ruder Boskovic Institute, Zagreb, Croatia

**Keywords:** Meteotsunami, GNSS, Atmosphere, Radio occultations, Total electron content

## Abstract

We study, for the first time, the physical coupling and detectability of meteotsunamis in the earth’s atmosphere. We study the June 13, 2013 event off the US East Coast using Global Navigation Satellite System (GNSS) radio occultation (RO) measurements, Sounding of the Atmosphere using Broadband Emission Radiometry (SABER) temperatures, and ground-based GNSS ionospheric total electron content (TEC) observations. Hypothesizing that meteotsunamis also generate gravity waves (GWs), similar to tsunamigenic earthquakes, we use linear GW theory to trace their dynamic coupling in the atmosphere by comparing theory with observations. We find that RO data exhibit distinct stratospheric GW activity at near-field that is captured by SABER data in the mesosphere with increased vertical wavelength. Ground-based GNSS-TEC data also detect a far-field ionospheric response 9 h later, as expected by GW theory. We conclude that RO measurements could increase understanding of meteotsunamis and how they couple with the earth’s atmosphere, augmenting ground-based GNSS TEC observations.

## Introduction

Meteorological tsunamis are destructive events causing casualties and significant property damage in coastal regions. They are generated by fast-moving mesoscale atmospheric disturbances that resonantly transfer energy from the air to the water bodies (Vilibić 2008). Their wave height is amplified if Proudman resonance is established (Proudman 1929), that is, when the propagation speed of the air disturbance, *U*, matches that of the wave, $c = \sqrt {gh}$, where *g* is the gravitational acceleration and *h* is the water depth. Close to the coast, harbor resonance could further amplify wave heights up to 2 orders of magnitude based on local bathymetry and coastal shape (Denamiel et al. 2018). The coupling at the air–water interface determines the generation and growth of meteotsunami waves, which are depending on the impacted location–largely driven by the propagation speed and period of the disturbance (Denamiel et al. 2020).

Meteotsunamis commonly occur in the Great Lakes region (Robertson et al. 2022), the Gulf of Mexico, the Atlantic coast, Japan, and the Mediterranean and Adriatic Seas (Rabinovich 2020), with ~ 3–10% of all historical tsunamis having meteorological origin. Kubota et al. (2021) studied the July 1, 2021, meteotsunami off the coast of northeastern Japan, traveling at about 110 m/s caused by a 0.5 ± 0.1 hPa atmospheric pressure disturbance. Rabinovich et al. (2020) studied the 15–20 cm meteotsunami on November 1, 2010, at the Canadian and US west coasts, which they found to have most likely been triggered by post-cyclonic activity the day before. Analysis of the derecho-driven meteotsunami recorded in the Great Lakes and along the US East Coast was used to quantify the contribution of air pressure versus wind disturbances in basins of different depths (Šepić and Rabinovich 2014). Shi et al. (2020) identified smaller than 20-cm meteotsunami waves during tropical cyclones, concluding that the rainbands of these mesoscale convective systems are responsible for their formation. Linares et al. (2019) showed that an atmospheric convective disturbance moving at 13–19 m/s caused 2.5–4.0 hPa pressure fluctuations, which triggered a 30-cm meteotsunami in Lake Michigan on July 4, 2003. Williams et al. (2019) simulated the June 23, 2016, 70-cm meteotsunami wave in the English Channel, reportedly triggered by a traveling precipitating convective system associated with 1–2.5 hPa surface pressure perturbations. Bechle et al. (2016) and Dusek et al. (2019) reported that the Great Lakes and the coastal USA experience on average about 100 0.3-m and approximately 25 1-m meteotsunami waves per year, respectively. Linares et al. (2016) found 11 meteotsunamis with waves in the 15–74 cm range in the Manistique River (MR) in Lake Michigan in a 2‐month period in summer 2012. Anderson et al. (2015) studied the generation mechanisms of the May 27, 2012, meteotsunamis on Lake Erie by modeling different hydrodynamic responses to atmospheric weather systems.

Despite their frequent occurrences and catastrophic impact, our knowledge about the underlying generation mechanisms for meteotsunamis is still poor, due to lack of observations at the required spatial–temporal scales and insufficient modeling of the associated small-scale traveling atmospheric disturbances (Belušić et al. 2007; Horvath and Vilibić 2014; Satake and Fujii 2014; Pattiaratchi and Wijeratne 2015; Denamiel et al. 2019). Tide gauges offer limited spatial coverage leading to large uncertainties in estimates of the meteotsunamis maximum wave heights (Vilibić et al. 2014). This also makes meteotsunamis difficult to detect at early stages in the open ocean due to the inability of the gauges to separate them from background occurring waves (Dunbar et al. 2017). The lack of high spatial resolution bathymetry measurements of regions of rapidly changing depth (Vilibić et al. 2016), especially at bays and harbors with large resonance factors (Denamiel et al. 2018), leads to misrepresentations of wave amplification. Vilibić et al. (2016) realized that tide gauges and sea level data alone are insufficient to address the abovementioned limitations and that no reliable early warning system currently exists.

Matoza et al. (2022) detected ionospheric TEC perturbations caused by traveling gravity waves (GWs) produced by the Tonga volcanic eruption, and Kubota et al. (2022), Omira et al. (2022), and Yamada et al. (2022) showed that these fast-moving GWs generated a meteotsunami wave with almost constant height of about 1.1 m up to 10,000 km away from the eruption site. Solovieva et al. (2021) reported the meteotsunami signatures in the earth’s ionosphere caused by a high-altitude dynamic system sweeping through the Mediterranean and Black Sea in June 2014. They captured the event in 1 min amplitude perturbations of VLF/LF signals from the ground-based International Network for Frontier Research on Earthquake Precursors (INFREP) network. Savastano et al. (2017) reported that the June 13, 2013, meteotsunami in the Atlantic Ocean, whose wave height fluctuated between 16 and 61 cm, perturbed the ionosphere by about 0.3 total electron content units (TECU, defined as 10^16^ el/m^2^) as observed by the nearby ground-based GNSS receiver network. Similar to tsunamis (Astafyeva 2019; Vergados et al. 2020), meteotsunamis trigger horizontally and vertically propagating gravity waves (GWs) that could reach the earth’s ionosphere. Nevertheless, ionospheric detection of meteotsunamis has not received as equal attention as tsunamigenic earthquakes (which exhibit prominent signatures in the earth’s electron density). Even lesser attention has been given to the study of how meteotsunamis dynamically couple with the lower troposphere, through which any tsunami-generated GW must travel on their way to the ionosphere.

The National Academy of Sciences (NAS) Decadal Survey (2018; Ch. 10) and the National Tsunami Hazard Mitigation Program (NTHMP) Strategic Plan [2018] explicitly identified the use of GNSS-derived products in advancing tsunami forecasting and assessment. Motivated by this recommendation and considering the abovementioned knowledge gaps, this study combines ground- and space-based GNSS observables and GW kinematics to investigate, understand, and characterize the meteotsunamis’ signature in the earth’s lower atmosphere and assess their detectability in the earth’s ionosphere. To the best of our knowledge, the impact of tsunamis has never been studied or observed in the neutral atmosphere observations, below 100 km altitude. This is an emerging field of study, and we envision that the preliminary results of this investigation will stimulate use of atmospheric data never considered before to help the natural hazards community in developing systems for early detection of open ocean meteotsunamis. The methodology section describes the processing techniques and the GNSS observables used. The results section presents and discusses our findings, and the conclusion section summarizes our analysis and provides recommendations.

## Methodology

**Fig. 1:**
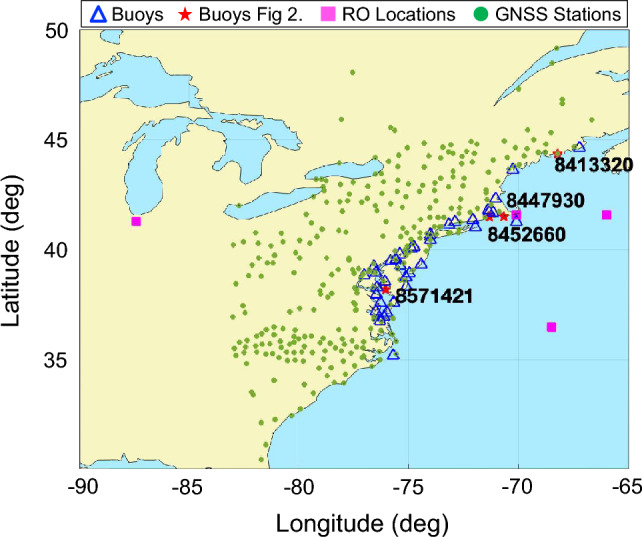
Geographic distribution of buoys (blue triangles), ground-based GNSS stations (green dots), and radio occultation (RO) soundings (magenta squares). Buoys marked in red asterisks and an 8-digit number are analyzed, and buoy #8,452,660 is collocated with an RO sounding

We study the June 13, 2013, meteotsunami off the eastward New Jersey coast, generated by a derecho traveling at 17–30 m/s inducing 3.5 hPa atmospheric pressure disturbances and maximum wave height of 60 cm (Wertman et al. 2014). We focus our analysis between 38 and 45  N and between 65 and 70  W from June 13, 2013 16:00 UTC onward. Table 1 summarizes our observational data, including product accuracy and spatial–temporal resolutions, and Fig. 1 presents the geographic distribution of all data sources used in this investigation. First, we identify collocations between GNSS radio occultation (RO) soundings and GW wavefronts generated by the traveling meteotsunami. We determine the thermodynamic properties and morphology of the neutral atmosphere from the planetary boundary layer up to 40 km at the collocation sites. Next, we use the RO temperature profiles to observationally determine normalized GW saturation curves in the troposphere and stratosphere.

**Table 1:** List of observational products and their associated characteristics

Instrument	Variable	Accuracy	Resolution	Altitude	Coverage
TIMED/SABER	Temperature	± 1.0–1.5 K	V: 2 km; H: 400 km	15–120 km	2002
GNSS RO	Temperature	< ± 0.5–1.0 K	V: 0.1 km; H: < 200 km	5–40 km	2000
Ground-based GNSS	Ionospheric TEC	< ± 0.03 TECU	N/A (vertically integrated)	~ 450 km	1998
Buoy stations	Sea level height	± 20 cm	In-situ measurements	sea level	1990

We compare them against theoretical estimates from linearized GW theory (Fritts and Alexander 2003) to identify the range of vertical wavenumbers in which the lower atmosphere is unsaturated (e.g., permitting the vertical wavelengths to traverse through the atmosphere). Next, we augment the RO measurements with collocated vertical temperature profiles from the Sounding of the Atmosphere using Broadband Emission Radiometer (SABER) instrument (Mertens et al. 2002) up to 100 km to try to trace the vertical propagation of the meteotsunami-generated GWs. Above the sporadic-E layer (altitude > 120 km) and up to 600 km, we could use ionospheric RO soundings to investigate the vertical coupling of the GWs in the earth’s ionosphere F region. However, the lack of collocated ionospheric ROs with the meteotsunami wavefront at the times of interest prevented us from performing such an analysis. Next, we use a dense ground-based multi-GNSS network of more than 300 stations tracking GPS, GLONASS, and Galileo navigation signals at 1, 5, 15, and 30 s rates to examine the state of the ionosphere before, during, and after the meteotsunami passing. Finally, we examine the time series of vertical total electron content (TEC) perturbations around the collocated RO and SABER soundings to assess the presence or absence of the meteotsunami-generated GWs.

### Radio occultation observation characteristics

RO is a limb-viewing remote sensing technique that measures the refraction of coherent L-band signals traversing the earth’s atmosphere between a GNSS satellite and a Low Earth Orbiter (LEO) that occults behind the earth’s limb (Kursinski et al. 1997). RO observations provide global tropospheric and stratospheric vertical temperature profiles with about 100–200-m vertical and less than 200-km horizontal footprint (which depends on the vertical resolution) up to 30 hPa with better than 0.5 K accuracy and about 0.05 K precision (Anthes et al. 2008; Schreiner et al. 2020). This makes RO observations an excellent observing platform of GWs with small ratios of vertical to horizontal wavelengths (Wu et al. 2006), such as those triggered by open ocean tsunamis. Gravity waves with vertical wavelengths smaller than 2 km are detectable in the lower atmosphere (Steiner and Kirchengast 2000; Lange and Jacobi 2003; Marquardt and Healy 2005). Here, we use the moist RO retrievals (wetPrf files) available at the COSMIC Data Analysis and Archive Center (CDAAC), which account for the water vapor contribution in the lower-to-middle tropospheric altitudes.

### Radio occultation horizontal footprint

The RO-retrieved temperatures at each tangent point represent the weighted-mean temperature over a horizontal distance along the L-band signal propagation path. Theoretically, aside from ionospheric error noise or horizontal inhomogeneities, this horizontal distance is a function of the vertical resolution, ${\Delta }H = 2\sqrt {2R{\Delta }Z}$ (Kursinski et al. 1997; Zeng et al. 2019) where *R* represents the earth’s local curvature at the tangent point and Δ*Z* is the vertical resolution. Typically, the vertical resolution is a function of the retrieval process of the RO profiles, and it ranges between 500 m and 1.5 km between the lower troposphere and middle atmosphere, respectively. However, recent advances in RO retrieval techniques (e.g., Canonical transform method (Gorbunov 2002)) yield profiles with a significantly better vertical resolution, and in this study, the CDAAC temperature profiles are provided at 100-m vertical resolution. In our case, and under ideal conditions, a vertical resolution of 100 m at 15-km altitude translates to a horizontal averaging of less than 100 km at the tangent point. Given that the RO weighting functions peak at the tangent point during an occultation (Kursinski et al. 2000), the majority of the atmospheric information represents the state of the atmosphere at the tangent point, with reduced contribution from within the horizontal footprint.

### Collocation technique between GW wavefronts, RO soundings, and SABER

We use historical RIFT (Real-time Inundation Forecasting for Tsunamis) model simulations (Wang et al. 2012) to follow the propagation (latitude, longitude, time) of the meteotsunami wavefront across the North Atlantic Ocean starting at 16:00 UT on June 13, 2013. The RIFT model is based on the finite difference discretization of the linear shallow-water equations. Next, we use the RO and TIMED/SABER geographic coordinates and the timestamp of the tangent points around the tropopause altitude (where the meteotsunami GW activity is expected to manifest) to calculate the spatial/temporal distance of the temperature soundings from the traveling meteotsunami-generated GWs. Using linearized GW theory, we estimate the vertical group velocities of the meteotsunami-generated GWs, which we use to compute the time required for the GWs to intersect the RO, the TIMED/SABER soundings, and the altitude of the ionosphere at which the electron density is maximum (hmF2). To first-order approximation, we assume that the GWs travel horizontally with the same speed as the meteotsunami.

### Background temperature determination and residual temperature estimation

All RO profiles are provided in a common vertical grid, equally spaced every 100 m, and we apply a low-pass second-order Savitzky–Golay filter (Savitzky and Golay 1964) with a 10-km window length to obtain the background temperature fit. The magnitude of the GW-induced temperature perturbations is estimated by differencing the background with the observed occultation profiles. Additionally, our filtering process removes background planetary wave activity, as their vertical wavelengths are much larger than 10 km at middle latitudes. We analyze the spectrum and vertical distribution of GW wavelengths by applying a Morlet wavelet on the temperature residuals. The magnitude of the residuals provides a metric of the coupling strength between the meteotsunami-generated GWs and the atmosphere. When analyzing TIMED/SABER vertical temperature profiles, we increase the window length to 20 km to avoid filtering out GWs whose vertical wavelength increases more than 10 km in the upper atmosphere.

### Buoy and ground-based GNSS station data processing

We process de-tided sea level measurements at 1 min interval from all available buoy stations along the US East Coast provided by NOAA’s National Data Buoy Center (NDBC). We bandpass filter the data in the 0.14–4.0 mHz frequency range to isolate traveling meteotsunami signatures in the GW spectrum. Also, we process RINEX files at 1, 5, 15, and 30 s intervals from more than 300 ground-based GNSS stations provided by NASA’s Crustal Dynamics Data Information System (CDDIS) (Noll 2010), NOAA’s Continuously Operating Reference Stations (CORS), and Scripps Orbit and Permanent Array Center (SOPAC) networks along the continental US East coast. We detrend the line-of-sight TEC links using polynomial fits up to degree 9 to remove long-duration trends and then bandpass filter the residuals in the same frequency range as the buoy data to focus on GW-induced ionospheric signatures. Finally, we convert the slant TEC to vertical TEC.

## Results and discussion

The June 13, 2013, meteotsunami was launched from the Delaware Bay and the coast of New Jersey (USA) and propagated eastward into the Atlantic Ocean. Upon reaching the edge of the continental shelf (about 135 km away from the coast), part of the meteotsunami energy was reflected back toward the US East coast, Nantucket Island, Long Island (NY), and Rhode Island (see, https://www.youtube.com/watch?v=ykABRe5Yt3c). We focus on the component of the reflected wave that travels northeastward toward Rhode Island. This is because we do not have neutral atmosphere GNSS RO observations over the states of Connecticut or New York to investigate the meteotsunami-atmosphere coupling over Long Island. The local bathymetry at the meteotsunami location at 18:30 UTC, traveling toward Rhode Island, ranges between 50 and 70 m (see, ncei.noaa.gov/maps/bathymetry), allowing for horizontal ocean wave speeds $c_{h} \approx \sqrt {gh}$ of 22–26 m/s. At 18:30 UTC, the RIFT model simulations showed meteotsunami wave heights of about 12 cm peak to peak with period of approximately 23 min (or *f*_wave_ ~ 0.7 mHz) and about 30 km horizontal wavelength. At such shallow waters, ocean waves with frequencies 0.3 < *f* < 3.0 mHz radiate their energy into the upper atmosphere via acoustic-gravity waves (Godin et al. 2015), making the meteotsunamis a continuous source of GWs.

**Fig. 2:**
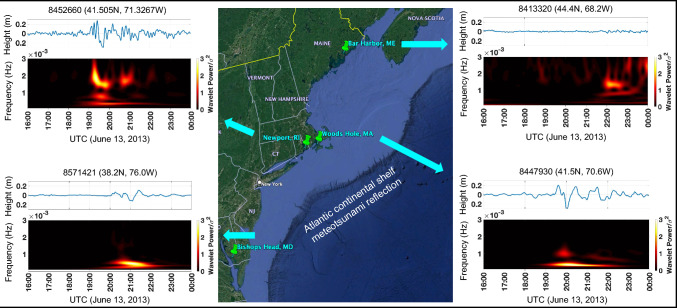
Sea-level height and normalized spectrograms as a function of time from 16:00 UTC until 23:59 UTC on June 13, 2013 for the four buoy stations marked with red asterisks in Fig. 1

An hour later, at 19:30 UTC, a 50 cm (peak-to-peak) wave was recorded at the Newport (Rhode Island) buoy station (8,452,660) at 41.5° N and 71.3° W, lasting for 2 h (see, Fig. 2). Wavelet analysis revealed wave frequencies in the GW range with dominant components between 1.5 and 3 mHz. The Woods Hole (Massachusetts) buoy station (8,447,930) at 41.5° N and 70.6° W registered similar magnitude sea level height fluctuations starting at 19:20 UTC and lasting for 3 h at 0.3–0.5 mHz frequency range that is consistent with RIFT model simulations. The meteotsunami waves arrived at Bishops Head (Maryland, 38.2° N, 76.03I° W) an hour later causing 30-cm peak-to-peak sea level height fluctuations at 0.1–0.5 mHz frequency range. West of the Bay of Fundy, across the coastline of Maine, the meteotsunami wave heights diminished to less than 10 cm (peak-to-peak), presenting dominant frequencies at 1.5 mHz.

**Fig. 3:**
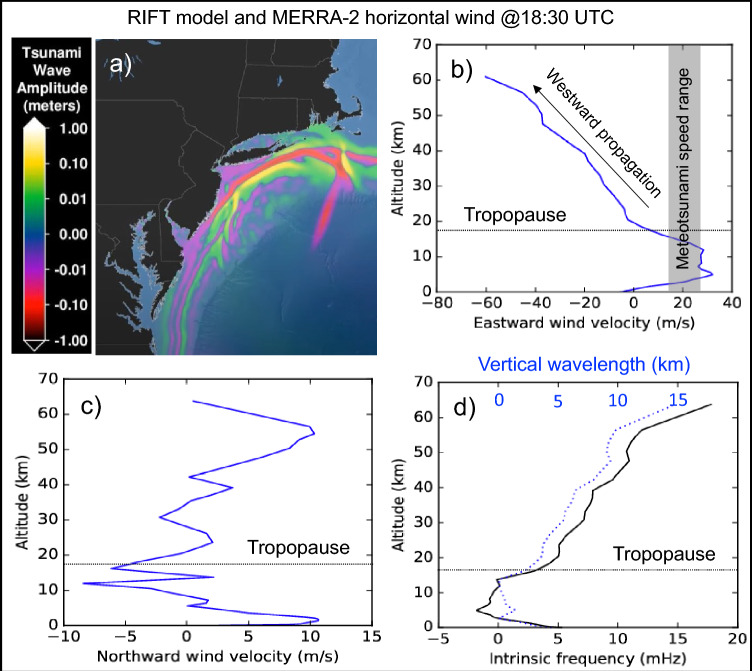
Snapshot of the June 13, 2013, meteotsunami together with wind conditions and GW properties at 18:30 UTC at the US coastal region. **a** Meteotsunami wavefront produced by the RIFT model (see, https://www.youtube.com/watch?v=ykABRe5Yt3c). **b**, **c** Vertical profiles of the eastward and northward wind components from MERRA-2, and **d** the estimated intrinsic frequency and vertical wavelength of anticipated meteotsunami-generated GWs

At 18:30 UTC, Modern-Era Retrospective analysis for Research and Applications v2 (MERRA-2) reanalysis (Gelaro et al. 2017) shows that from the top of the planetary boundary layer up to 17 km, the background horizontal wind speed matches that of the meteotsunami wavefront (see, Fig. 3b and c). The intrinsic frequency, $\hat{\omega } = \omega - k_{x} \overline{u} - k_{y} \overline{\upsilon }$ where ($\overline{u}$, $\overline{\upsilon }$) is the mean background wind and ($k_{x}$, $k_{y}$) the horizontal wavenumbers, of the meteotsunami-induced GWs becomes comparable to the Coriolis force (see, Fig. 3d; solid black line) causing GWs to travel over large horizontal distances from their generation source at ~ 22–26 m/s (Eckermann 1992). The theoretical magnitude of the meteotsunami-induced GWs’ vertical wavelength, *λ*_*z*_, is less than 2 km at tropopause growing up to 15 km in the lower mesosphere (see, Fig. 3d; dotted blue line), suggesting that in the troposphere we should not expect noticeable GW activity. Given an average $c_{h} \approx$ 24 ± 2.0 m/s, GWs triggered at 18:30 UTC could travel as far as 120–140 km northward within 90 min, while vertically propagating with $c_{v} \approx 3.0$ m/s (estimated using GW linear theory).

**Fig. 4:**
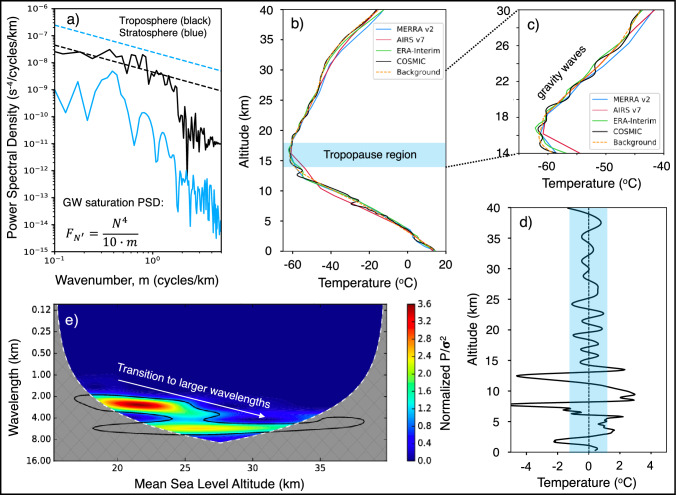
An example of the atmospheric response to the meteotsunami at 18:30 UTC captured by multiple space-based observing platforms together with the anticipated GW properties. **a** Observed vertical wavenumber spectra in the troposphere (solid black) and stratosphere (solid blue) and theoretical saturation lines (dashed). **b** Vertical temperature profiles from MERRA-2 (blue), AIRS v7 (red), ERA-Interim (green), COSMIC (black), and the background fit (dashed orange). **c** Zoom-in of vertical temperature profiles in the 14–30 km altitude range, **d** vertical temperature fluctuations, and **e** normalized wavelet power with respect to its variance of the temperature residuals in panel (**d**) between 15 and 40 km

At 42.2N and 70.3W at 19:54 UTC, about 140 km NE and after 90 min where the meteotsunami-induced GWs are expected to be, a COSMIC spacecraft was vertically scanning the atmosphere every 100 m. The COSMIC temperature profile reveals that the troposphere allows the vertical propagation of GWs with vertical wavenumbers *k*_*z*_ of about 0.1–5.0 cycles/km, as the slope of the theoretical saturation line matches the observed (see, Fig. 4a; solid and dashed black lines). This indicates that meteotsunami-induced GWs can traverse the troposphere and enter the stratosphere (above 18 km), which is unsaturated to *k*_*z*_ < 1.0 cycles/km (see, Fig. 4a; blue lines). The RO vertical temperature profile captured distinct wave-like activity above 14 km (see, Fig. 4b, c). However, these features are missed by spatially and temporally collocated AIRS v7 (Thrastarson et al. 2021), ERA-Interim (Dee et al. 2011), and MERRA-2 (Gelaro et al. 2017) datasets, due to their coarse vertical resolution that smears out small-scale vertical structures. Removing the background variability, we extract vertical oscillations with ± 2–3 K (peak-to-peak) amplitude (see, Fig. 4d) throughout the stratosphere. A Morlet wavelet analysis of the temperature residuals in the lower stratosphere shows vertical oscillations with *λ*_*z*_ about 2–3 km, which match the theoretical estimates in Fig. 3d (dashed blue) below 25 km. In the middle-to-upper stratosphere, the vertical wavelength increases to *λ*_*z*_ ~ 5–6 km (see, Fig. 4e) also agreeing with expected values in Fig. 3d (dashed blue) above 25 km. Additionally, model simulations of low-frequency GWs showed that the meteotsunami at 18:30 UTC with 10-cm amplitude, 23-min period, and 30-km horizontal wavelength heading 31.65° toward RI could cause ± 1.5 K temperature perturbations above 15 km supporting the RO observations.

**Fig. 5:**
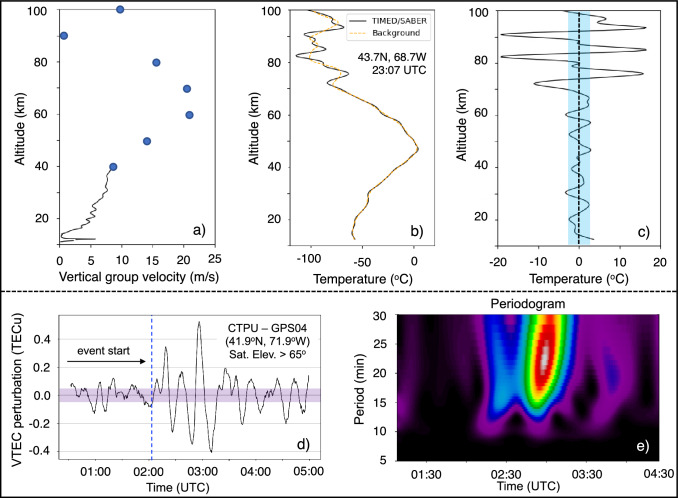
Signatures of meteotsunami-induced gravity waves (GWs) in the neutral atmosphere and ionosphere. We quantify their 3D properties as a function of time and altitude. **a** Estimated vertical group velocity from COSMIC (black) and TIMED/SABER observations (blue circles) with altitude. **b** Vertical temperature profile from TIMED/SABER (black) with the background fit (dashed orange), and **c** estimated vertical temperature fluctuations. **d** Time series of vertical TEC perturbations over station CTPU (black) with its precision level (purple rectangle), and **e** periodogram of the vertical TEC perturbations with time

Given a horizontal and vertical meteotsunami wave propagation speed of $c_{h} \approx 24$ m/s and $c_{v} \approx 3$ m/s, we estimate that GWs launched around 18:30 UTC at the sea surface (see, Fig. 3a) are expected to reach the upper atmosphere at 43.7° N and 68.7° W in 4.5 h at approximately 23:11 UTC. We confirmed the estimated travel time by calculating the ratio of the vertical to horizontal group velocity, $\left| {c_{v} /c_{h} } \right| = \left( {\hat{\omega }^{2} - f^{2} } \right)^{1/2} /N$ using satellite observations. We used the Horizontal Wind Model 2014 (HWM2014) and the TIMED/SABER temperature observations to compute the intrinsic frequency ($\hat{\omega }$), the Brunt–Väisälä frequency (*N*), and the vertical group velocity of the GWs from 40 up to 100 km at the RO location (see, Table 2 and Fig. 5a).

**Table 2:** Vertical group velocity estimations and their travel times between altitude levels

Altitude (km)	${\hat{\omega }}$(mHz)	c_v_ (m/s)	Travel time (min)
40 km → 50 km	7.90	8.70	19.2
50 km → 60 km	10.1	14.1	11.8
60 km → 70 km	13.3	21.0	7.90
70 km → 80 km	14.7	19.1	8.70
80 km → 90 km	12.9	16.4	10.1
90 km → 100 km	1.10	1.10	151.5

We estimated the travel time of GWs at each altitude and found that GWs shown in the RO observations at 19:54 UTC will reach the upper atmosphere in less than 3 h and 29 min that is before 23:23 UTC. Coincidentally, at 23:07 UTC, the TIMED/SABER satellite (orbit #: 62,385 and event #: 38) was scanning the atmosphere from 10 to 100 km and presented distinct wave activity (see, Fig. 5b). Applying a Savitzky–Golay filter with 20-km window size on the observed temperature profile, we extract temperature residuals as described in Sect. “Background temperature determination and residual temperature estimation.” We identify distinct vertical oscillations accompanied with temperature perturbations that match those observed by the RO observations in the 20–40-km altitude range. Above 50 km, the vertical wavelength increases growing to *λ*_*z*_ ~ 10–12 km in the middle and upper mesosphere causing peak-to-peak ± (15–20) K temperature perturbations (see, Fig. 5c). Using HWM2014 and $N\approx 0.02$ Hz from 110 km up to hmF2, located at about 280 km based on the International Reference Ionosphere 2016, we estimate that GWs would arrive at hmF2 before 02:50 UTC on June 14, 2013.

Interestingly, at 41.9° N and 71.9° W at the GNSS station CTPU on June 14, 2013, we observed an enhancement of the background ionospheric vertical TEC starting at 02:00 UTC that lasted for about an hour (see, Fig. 5d). The magnitude of the observed vertical TEC perturbation ranged between 0.4 and 0.8 TECU (peak-to-peak), exhibiting a periodic behavior of 15–25 min (see, Fig. 5e), falls within the reported meteotsunami wave period at 18:30 UTC.

**Fig. 6:**
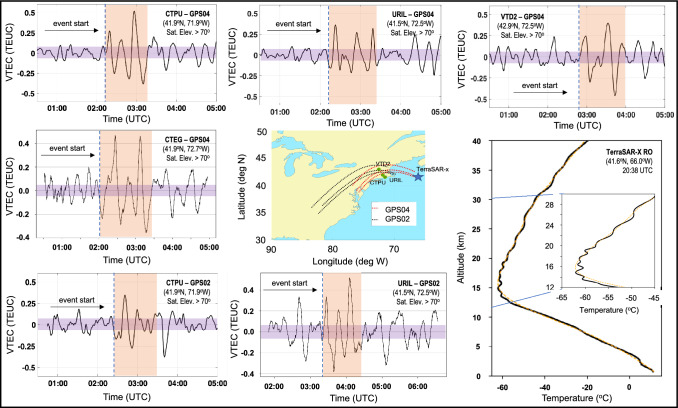
Time series of vertical TEC perturbations over GNSS stations CTPU, URIL, and CTEG tracking GPS satellites pseudorandom noise (PRN) 04 and 02. Purple rectangles mark the typical vertical TEC noise level and the dashed blue lines mark the start of the vertical TEC enhancement. (Bottom right) TerraSAR-X RO vertical temperature profile (black) and the background fit (orange) at 20:38 UTC

Numerous close-by GNSS stations tracking multiple GPS satellites also captured similar vertical TEC enhancements at, or around, 02:15 UTC (see, Fig. 6) supporting the signal over CTPU station in Fig. 5d. GNSS stations URIL, CTPU, and CTEG are located within 200 km from the Rhode Island and Massachusetts buoy stations, except from VTD2 which is more than 250 km away. We showed that the total travel time up to hmF2 of meteotsunami-induced GWs launched at 18:30 UTC south of the Nantucket Island is about 9 h. This suggests that vertically propagating GWs are expected to be observed in the ionosphere prior to 03:00 UTC on June 14, 2013. We focused on the ionosphere within 200 km radial distance from the GNSS stations (to minimize contamination of the signal from far-field ionospheric disturbances) and identified two GPS satellites (PRNs 02 and 04) whose azimuthal track is perpendicular to the traveling meteotsunami (see, Fig. 6).

The satellite-station links CTPU-GPS04, CTEG-GPS04, and URIL-GPS04 show simultaneous vertical TEC enhancements at 02:15 UTC with peak-to-peak amplitudes of 0.5–0.8 TECU. The signal appears to have a 20 min period and lasts for about an hour. VTD2 also captures vertical TEC enhancement of similar magnitude and period a few minutes later, at 02:50 UTC, being further away from the rest of the stations. The purple rectangle area marks the vertical TEC noise level indicating that perturbations prior to the enhancement are statistically insignificant. Also, CTPU and URIL stations tracking a different GPS satellite (PRN 02) show simultaneous vertical TEC enhancement around 02:30 UTC with similar perturbation amplitudes and periods as when tracking GPS satellite 34. Capturing the vertical TEC enhancement at different azimuth and by different station-satellite pairs (see, Fig. 6) provides stronger evidence of the meteotsunami-induced GW detection in the ionosphere.

Apart from the ground-based GNSS measurements, the TerraSAR-X satellite carrying a GNSS RO receiver also captured small-scale GW activity (see, Fig. 6; bottom right). The meteotsunami wavefront at 18:30 UTC could not have caused the observed GW in TerraSAR-X. However, the local bathymetry at the Atlantic continental slope, south of the Nantucket Island, where the meteotsunami is reflected northeast toward the Georges Bank (a large elevated area of the sea floor between Cape Cod, Massachusetts, USA, and Cape Sable Island, Nova Scotia, Canada) at 17:57 UTC, is on average 100 m (https://www.ncei.noaa.gov/maps/bathymetry). This suggests that meteotsunami-induced GWs launched over the continental slope would travel northeast at about 31 m/s, reaching the TerraSAR-X RO site (320 km away at 41.6° N and 66° W) within 2 h and 50 min, at approximately 20:49 UTC.

**Fig. 7:**
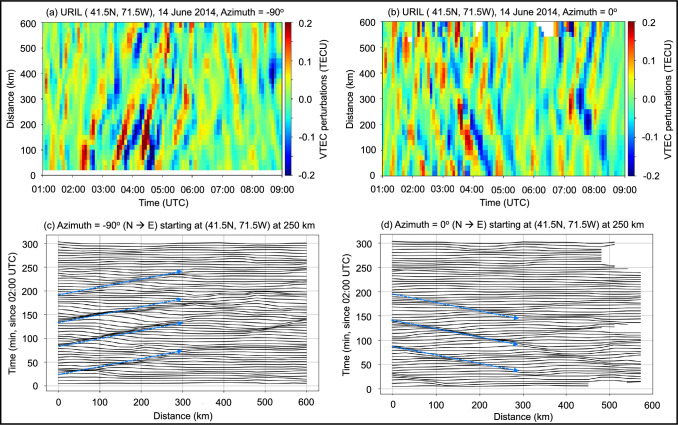
Keograms of ionospheric vertical TEC perturbations as a function of time on June 14, 2013, from 01:00 UTC until 09:00 UTC and distance centered over the URIL station at **a** −90° and **b** 0° azimuths, respectively. Coherent ionospheric vertical TEC perturbations at 250 km as a function of distance and time over the URIL station at **c** −90° and **d** 0° azimuths, respectively

With multiple stations presenting similar ionospheric TEC signatures, we computed the detrended ionospheric LoS TEC perturbations from all available station-satellite links over the continental US. We projected the ionospheric TEC to a fixed altitude of 250 km (which coincides with the hmF2), binned them in a 0.15 × 0.15 grid, and smoothed them with a 5 × 5 Gaussian Kernel (Vadas and Azeem 2021) to study the ionospheric variability. Focusing over the US East Coast, over the URIL station, we observed distinct wavefronts of traveling ionospheric disturbances (TID). We estimated the TID 2D horizontal wavelength, *λ*_*H*_, and phase speed, *C*_*H*_, from the ionospheric TEC map along a constant latitude at 41.5° N for zonal components (*λ*_*x*_, *c*_*x*_) and along a constant longitude at 71.5° W for meridional components (*λ*_*y*_, *c*_*y*_). The wavelength components were inferred from a spectral analysis of the respective Keograms (Mrak et al. 2018) (see, Fig. 7a and b), and the phase speed components were estimated from the slope diagram following coherent TEC perturbations with time (see, Fig. 7c and d). We found *λ*_*x*_ = 285 km, *λ*_*y*_ = −200 km, *c*_*x*_ = −91 m/s, *c*_*y*_ = −100 m/s, which correspond to horizontal wavelength and phase velocity of *λ*_*H*_ = 164 km and *C*_*H*_ = 67.3 m/s, respectively.

## Conclusions

We assumed that meteotsunamis couple with the earth’s atmosphere via low-frequency GWs as their intrinsic frequency is comparable to the Coriolis frequency. Thus, we hypothesized that meteotsunamis would have a delayed atmospheric response at far-field, due to their small vertical-to-horizontal group velocity ratio. To test our hypothesis, we identified spatially and temporally collocated vertical temperature profiles with the reflected meteotsunami. We detected small-scale GW activity in the stratosphere and mesosphere 1.5 and 5.0 h after the meteotsunami wavefront, located south of the Nantucket Island, at distances greater than 150 km and 200 km, respectively. We found that the times and distances of the observed GWs were closely related to theoretical estimations of low-frequency GWs tracing, supporting our hypothesis. Our initial results revealed that the background horizontal wind field drives the physical coupling between meteotsunamis and the earth’s atmosphere by modulating the GWs’ intrinsic frequency, and thus their 3D spatial propagation. We found that meteotsunamis cause far-field enhancements of the background ionospheric vertical TEC about 9 h after onset with periods that match that of the meteotsunami wavefront. To understand the dynamic coupling of the meteotsunami-atmosphere–ionosphere system requires detailed investigation, which is different from that of open ocean high-frequency tsunamis of a seismic origin. Despite detecting potential meteotsunami-generated GWs in Figs. 5d and 6, we still do not understand how such GWs interact with the earth’s ionosphere as a function of altitude. One could use high vertical resolution (about 1–2 km) ionospheric RO soundings (Yue et al. 2014), where GWs would manifest as electron density perturbations, to understand how tsunamis and meteotsunamis couple with the ionosphere and extract their wave properties. Even though the meteotsunami-generated GWs do not arrive in the ionosphere until after 9 h from the event, the ionospheric ROs would provide an observational constraint to the horizontally propagating ionospheric disturbances measured by ground-based GNSS stations.

We hope that this preliminary analysis will spur the community’s curiosity in studying the atmospheric and ionospheric response and detectability of meteotsunamis in light of mitigating societal needs. Due to the delayed response of the earth’s ionosphere, we believe that near-real time ground-based GNSS-TEC measurements might not be an ideal remote sensing technique in the early detection of meteotsunamis. Even if we found an atmospheric signature of meteotsunamis in RO soundings, the lack of globally available near-real time occultations prevents continuous monitoring. Currently, there is no known method that can be used to issue meteotsunami early warnings, despite the availability of tsunami early warning systems. We argue that near-real-time spatially dense and globally available ROs could augment existing GNSS-TEC-based tsunami detection methods (Martire et al. 2023) and increase coverage over unmonitored oceanic regions. Although there are multiple steps in analyzing meteotsunami signatures in the neutral atmosphere and ionosphere, the methodology and data processing steps outlined in this study can be easily implemented in tsunami detection systems. In the future, only if GNSS ROs became available at near-real time with better spatiotemporal coverage, we could be able to monitor the atmosphere and detect the occurrence of tsunamis and meteotsunamis by analyzing the properties of GWs associated with the events and separate them from the background GW activity. Key to the success of our methodology lies on the fact that we need to follow the meteotsunami-generated GWs from their source continuously up the ionosphere, in order to increase the detection probability and minimize false positives.

## Data Availability

All data sources are publicly available, and all weblinks provided here are currently accessible. We downloaded the radio occultation (RO) temperature profiles from the COSMIC Data Analysis and Archive Center (CDAAC) available at: https://cdaac-www.cosmic.ucar.edu. We downloaded the TIMED/SABER temperature profiles from: http://saber.gats-inc.com/browse_data.php. We used the online Horizontal Wind Model version 2014 (HWM2014) to retrieve vertical profiles of the horizontal wind components from: https://ccmc.gsfc.nasa.gov/models/HWM14~2014/. We downloaded the RINEX files for all the ground-based GNSS stations from NOAA’s Continuously Operating Reference Stations (CORS): https://geodesy.noaa.gov/CORS/, from the Scripps Orbit and Permanent Array Center (SOPAC): http://sopac-csrc.ucsd.edu/, and from the Crustal Dynamics Data Information System (CDDIS): https://cddis.nasa.gov/archive/gnss/data/daily/. We downloaded the sea level height buoy time series from NOAA’s National Data Buoy Center (NDBC) available at https://www.ndbc.noaa.gov/. We downloaded the AIRS v7 and MERRA-2 temperature profiles from: https://disc.gsfc.nasa.gov/.
